# Efficacy and mechanisms underlying MRI-guided HD-tDCS combined with aerobic exercise to ameliorate cognitive impairment associated with schizophrenia

**DOI:** 10.3389/fpsyt.2026.1742634

**Published:** 2026-02-24

**Authors:** Yange Wei, Zengyuan Shen, Peng Luo, Shanyuan He, Hanshuo Su, Rongxun Liu, Yanran Wu, Juan Wang, Jingdan Zhang, Guangjun Ji, Fei Wang, Chuansheng Wang

**Affiliations:** 1 Department of Early Intervention, Mental Health and Artificial Intelligence Research Center, The Second Affiliated Hospital of Xinxiang Medical University, Henan Mental Hospital, Xinxiang, China; 2 NHC Key Laboratory of Mental Health (Peking University), Peking University Sixth Hospital, Peking University Institute of Mental Health, National Clinical Research Center for Mental Disorders (Peking University Sixth Hospital), Beijing, China; 3 School of Public Health, Xinxiang Medical University, Xinxiang, China; 4 Unit of Early Intervention, The Affiliated Brain Hospital of Nanjing Medical University, Nanjing, China; 5 Department of Psychiatry, Yale School of Medicine, New Haven, CT, United States

**Keywords:** aerobic exercise, cognitive impairment associated with schizophrenia, high-definition transcranial direct current stimulation, schizophrenia, study protocol

## Abstract

**Background:**

The primary treatment for schizophrenia currently relies on medication. Nevertheless, the efficacy of medication for Cognitive Impairment Associated with Schizophrenia (CIAS) is constrained, and it is also accompanied by side effects. Consequently, the investigation of novel non-pharmacological strategies is essential. High-definition transcranial direct current stimulation (HD-tDCS) and aerobic exercise (AE) have emerged as promising approaches for cognitive enhancement in individuals with schizophrenia. This study aims to evaluate the efficacy of integrating HD-tDCS with AE for CIAS and to elucidate the underlying mechanisms of this synergistic intervention.

**Methods:**

A randomized, double-blind, controlled trial will be conducted. The CIAS will be randomly allocated to one of four groups: MRI-guided HD-tDCS + AE, MRI-guided HD-tDCS alone, AE alone, and a control group. Structural magnetic resonance imaging (MRI) data will be obtained to determine the optimal electrode placement. The central electrode will be positioned over the medial prefrontal cortex (mPFC). Both HD-tDCS and AE will be administered five times per week over a four-week period, resulting in a total of 20 sessions. The primary outcome measure will be the change in cognitive function, evaluated using the MATRICS Consensus Cognitive Battery. Secondary outcomes will include changes assessed by the Repeatable Battery for the Assessment of Neuropsychological Status and the Wisconsin Card Sorting Test which are designed to evaluate global and executive functions. The Facial Emotion Perception Test and the Voice Emotion Perception Test will be utilized to assess social cognition. The severity of clinical symptoms will be quantified through the Positive and Negative Syndrome Scale and the Brief Psychiatric Rating Scale. This study will incorporate functional near-infrared spectroscopy, MRI, electroencephalography, P300 event-related potential, eye movement examination and plasma brain-derived neurotrophic factor (BDNF) levels to investigate the underlying mechanisms. Assessments will be evaluated at baseline (T0), after 2 weeks (T1), after 4 weeks (T2), and after 6 months (T3).

**Discussion:**

The integration of MRI-guided HD-tDCS targeting the mPFC and AE presents an efficacious and individualized treatment strategy for CIAS. This proof-of-concept study may provide a multi-dimensional view of biological mechanisms underlying HD-tDCS combined with AE in precision psychiatry.

**Trial registration details:**

The study is registered with https://www.chictr.org.cn/ protocol registration number ChiCTR2500106980 (date of registration: 1. August. 2025). It was approved by the Research Ethics Committee of the Second Affiliated Hospital of Xinxiang Medical University (Approval Code: XYEFYLL-2025-16, Approval Date: 17 February 2025). Recruitment began in December 2025.

## Background

Cognitive Impairment Associated with Schizophrenia (CIAS) is a core symptom of schizophrenia, and its severity directly affects patients’ social functioning, activities of daily living, and long-term prognosis (1, 2). The extent of cognitive function improvement is a key prognostic indicator for patients with schizophrenia (3). Current antipsychotic medications primarily target the dopamine system and effectively alleviate positive symptoms but have limited impact for CIAS (4). Cognitive abilities of individuals with schizophrenia do not necessarily enhance with the remission of psychotic symptoms (5, 6). Although several potential cognitive-enhancing agents have advanced to Phase III trials as adjuncts to antipsychotic treatment, none have yet demonstrated sufficient efficacy to secure FDA approval for schizophrenia (7). Furthermore, variability in individual responses and adherence to therapy pose additional challenges (8). Consequently, the exploration of novel non-pharmacological strategies is critically important.

High-definition transcranial direct current stimulation (HD-tDCS) is a non-invasive intervention technique that utilizes a constant direct current to modulate cortical neuron activity. Compared to conventional tDCS, HD-tDCS provides more precise cortical stimulation targeting, enhanced cortical penetration, and the potential for targeted neuromodulation, which can lead to specific symptomatic changes (9, 10). Multiple randomized controlled trials have indicated that tDCS targeting regions such as the prefrontal cortex significantly enhances working memory and executive functions in patients (11, 12). Research has demonstrated that HD-tDCS can improve the integrity of specific white matter tracts in individuals with chronic schizophrenia, which is associated with enhanced attentional function (13). A recent meta-analysis further substantiates that HD-tDCS exerts a moderate effect on overall cognitive function in schizophrenia (14). Mechanistically, HD-tDCS may enhance cognition by modulating cortical neuronal excitability, promoting synaptic plasticity, and influencing associated neurotransmitter systems (15). Overall, HD-tDCS exhibits clear potential and value for CIAS. Concerning the selection of stimulation sites, previous studies have demonstrated that cognitive impairments, such as deficits in episodic and working memory and challenges in emotional regulation, are associated with dysfunction in the medial prefrontal cortex (mPFC) and altered connectivity with subcortical regions (16). Furthermore, dysregulation of dopaminergic activity in the mPFC is associated with the progression of schizophrenia (17, 18). Delayed dopamine release in the medial prefrontal cortex (mPFC) is a defining characteristic of the pathophysiology of schizophrenia (19). A deficiency in the dopamine transporter leads to reduced spine density in mPFC pyramidal neurons, resulting in mPFC dysfunction and potentially contributing to behavioral abnormalities in schizophrenia. The primary manifestations are abnormalities in attention (e.g., poor concentration), memory (especially working memory), and executive functions (e.g., deficits in planning, decision-making, and cognitive flexibility) (20). Postmortem studies and schizophrenia mouse models, induced by neonatal basolateral amygdala lesions, also demonstrate reduced dendritic spine density in mPFC pyramidal neurons (21, 22). Consequently, the mPFC has been selected as the target in this study. Research utilizing HD-tDCS targeting the mPFC in schizophrenia remain limited. Further, individual anatomical variations can substantially affect the outcomes of HD-tDCS altering the distribution of electrical currents within the cortical regions, thereby impacting therapeutic efficacy (23, 24). The use of MRI-guided HD-tDCS interventions enables the identification of optimal electrode placements tailored to each individual, thereby reducing the impact of structural brain differences and enhancing the effectiveness of HD-tDCS (25, 26). Consequently, this study will utilize MRI to obtain anatomical data for each participant, and develop appropriate therapeutic target for individualized precision therapy for CIAS.

Aerobic exercise (AE) is one of the psychosocial interventions with most robust evidence for CIAS (27). AE exerts its effects through multiple mechanisms. It has been demonstrated to influence brain neuroplasticity via neurogenesis and structural alterations (28, 29). Furthermore, AE can mitigate age-related cognitive decline by altering brain metabolism, structure, and connectivity (30). Additionally, AE significantly enhances the activity of mPFC and improves cognitive function within this region (31, 32). Accumulating evidence suggests that structured AE programs can lead to improvements in executive function, attention, and memory in patients with schizophrenia, possibly through mechanisms involving enhanced prefrontal oxygenation and neurotrophic support (33). HD-tDCS has demonstrated promising results in disease intervention, recent studies indicate that its therapeutic effects may be further enhanced when combined with AE intervention methods (34, 35). Consequently, this research aims to examine the synergistic effects of combined HD-tDCS with AE for CIAS.

This study employed a simultaneous intervention approach utilizing HD-tDCS and AE, based on the evidence of the temporal effect of combined intervention. A comprehensive review suggests that integrating neuroregulation with psychosocial methods yields superior outcomes in enhancing cognitive function and alleviating negative symptoms in individuals with schizophrenia, compared to singular interventions; it also highlights the critical role of intervention timing in determining efficacy (36). Furthermore, a randomized controlled trial further confirmed that the concurrent application of neuroregulation and psychosocial strategies significantly ameliorates cognitive deficits and negative symptoms in schizophrenia patients (37). Consequently, the synchronous research design is underpinned by a sound theoretical and empirical rationale. In terms of the therapeutic mechanism, HD-tDCS and AE may exert their effects through a multi-level synergies: AE enhances the secretion of brain-derived neurotrophic factor (BDNF), thereby promoting synaptic plasticity (38, 39), BDNF is not only a key factor for the survival and functional maintenance of neurons, but also regulates synaptic structure and function, especially in the prefrontal cortex, where it supports the long-term potentiation effect, which is the neural basis for learning and memory formation (40). HD-tDCS can regulate the excitability of cortical neurons through direct current, and it supports the neuroplastic mechanism mediated by BDNF, thereby forming a bidirectional synergistic enhancement effect (41). Secondly, AE improves the blood flow to the brain, reduces oxidative stress responses, and enhances mitochondrial function and metabolic efficiency, thereby providing a more favorable physiological environment for the brain (42). Concurrently, HD-tDCS directly modulates the excitability of the mPFC, enhancing the metabolic improvement such as reduced oxidative stress and increased cerebral blood flow induced by AE, thus facilitating cognitive enhancement (43). The combination of the two can promote the increase of blood flow and oxygenation levels in the mPFC area, thereby enhancing its role in executive functions, working memory, and emotional processing. Exploring this integrated intervention approach could offer a novel and effective intervention option for CIAS.

This study aims to investigate three principal aspects. The primary objective of this study is to evaluate the efficacy of the combined intervention of HD-tDCS and AE in enhancing cognitive function at the individual level. Additionally, we aim to assess the long-term impact of this intervention over a six-month period, focusing on changes in cognitive function, clinical symptoms-measured by the Positive and Negative Syndrome Scale (PANSS) and Brief Psychiatric Rating Scale (BPRS), social function-measured by Social Disability Screening Schedule (SDSS), and Schizophrenia Quality of Life Scale (SQLS). Furthermore, the study seeks to elucidate the biological mechanisms of MRI-guided HD-tDCS and AE from a multi-dimensional perspective. By evaluating the feasibility and safety of this precision approach, the study aims to provide biological insights into individualized treatment strategies for CIAS.

## Methods

### Study design

**Figure 1:**
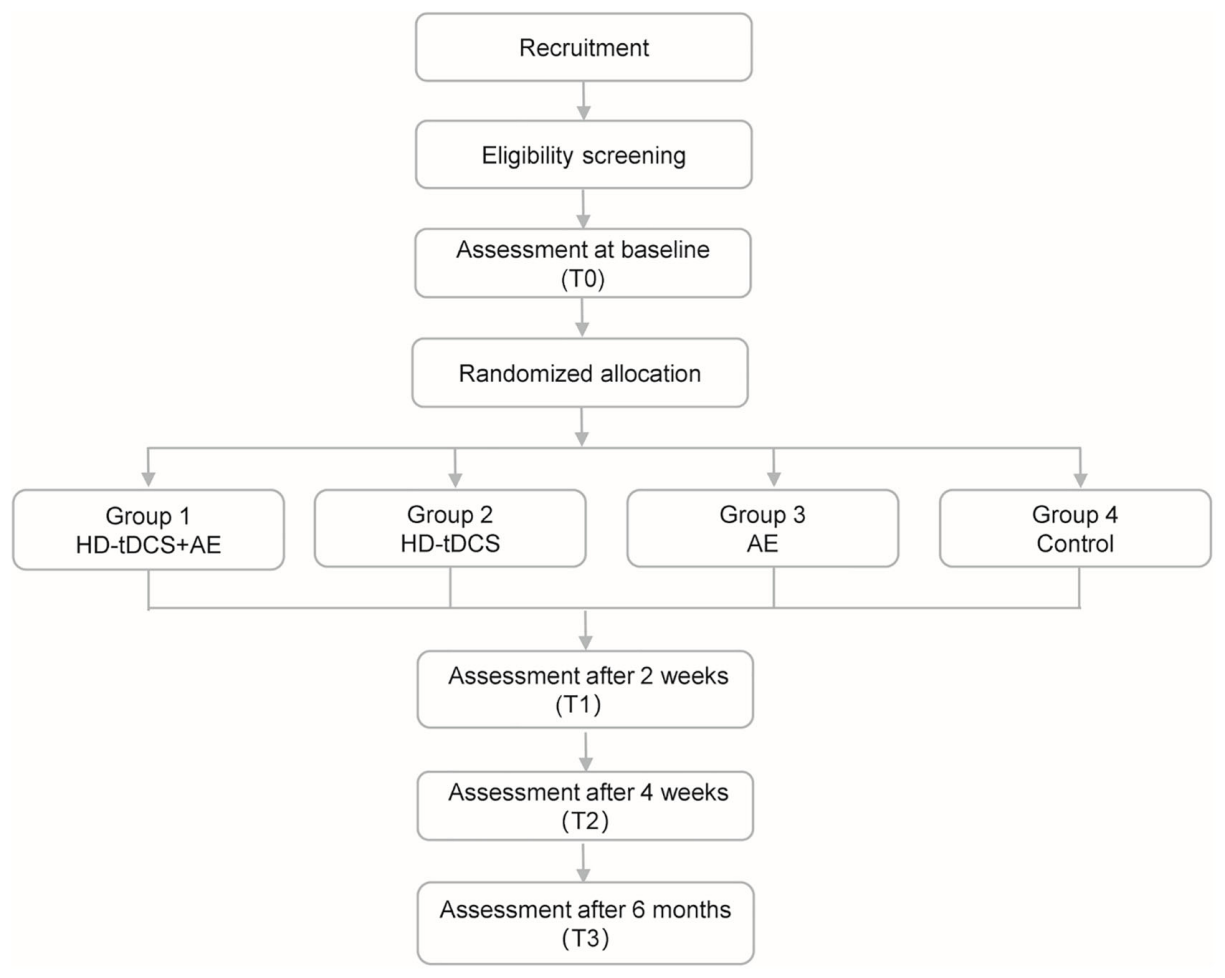
Flowchart of the study design.

This randomized, double-blind, controlled clinical trial evaluates the efficacy of combining HD-tDCS with AE to improve cognitive function in individuals with CIAS. Eligible participants will be randomly assigned to one of four groups in equal proportions (1:1:1:1): MRI-guided HD-tDCS +AE (Group 1), MRI-guided HD-tDCS alone (Group 2), AE alone (Group 3), and a control group (Group 4). Assessments will be conducted at baseline (T0), at 2 weeks (T1), at 4 weeks (T2), and at 6 months post-intervention (T3). The specific intervention measures for each group are delineated as follows: the MRI-guided HD-tDCS + AE group first undertakes a 15-minute warm-up exercise. Subsequently, HD-tDCS and AE treatments are administered concurrently for a duration of 30 minutes. The HD-tDCS group receives 30 minutes of electrical stimulation per session. The AE group’s intervention comprises a 15-minute warm-up followed by a 30-minute formal AE stage, amounting to a total of 45 minutes. The control group does not receive HD-tDCS or AE but instead is provided with routine psychiatric care, including regular psychiatric follow-ups, medication management, and general health guidance, but no experimental interventions. Each intervention for the aforementioned groups is conducted once daily, five days a week, over a period of four weeks, culminating in a total of 20 sessions. The study design is illustrated in **Figure 1**.

### Recruitment

Participants will be recruited from the Department of Psychiatry at the Second Affiliated Hospital of Xinxiang Medical University. The trial will be publicized via the hospital’s official website and various media outlets. Information leaflets will be distributed within the Department of Psychiatry, where psychiatrists will provide an overview of the study. Potential participants will receive both oral and written information regarding the research procedures, along with their potential benefits and risks. All participants and their legal guardians, will sign informed consent forms. In accordance with the Helsinki Declaration on Ethical Principles for Medical Research Involving Human Subjects, this study received approval from the Research Ethics Committee of the Second Affiliated Hospital of Xinxiang Medical University (Approval Number: XYEFYLL-2025-16, Approval Date: February 17, 2025) and has been registered with the Chinese Clinical Trial Center (Registration Number: ChiCTR250010698). The trial will take place at the Second Affiliated Hospital of Xinxiang Medical University. This research protocol adheres to the 2013 Standard Protocol Items: Recommendations for Intervention Trials (SPIRIT) Statement Guidelines (44).

### Eligibility criteria

Eligible individuals must meet the following inclusion criteria (1): The current episode of the patient aligns with the diagnostic criteria for schizophrenia according to the Diagnostic and Statistical Manual of Mental Disorders-5 (DSM-5) (2). patients who achieved the stable period through oral antipsychotic drug treatment, as judged by the following criteria: delusion, hallucinatory behaviors, exaggeration, and suspicion/victimization items in the positive and negative symptom scale (PANSS), abnormal thought content scores of ≤ 5 in the general psychopathology scale, and PANSS conceptual disorganization scores of ≤ 4, if all the aforementioned criteria were met, the patient was considered to be in a stable period of schizophrenia (45) (3). Patient must be undergoing treatment with atypical antipsychotic medications, with equivalent doses calculated using the defined daily dose method (4). To minimize study population heterogeneity only Han ethnic participants will be enrolled (5). Aged between 18 and 55 years (6). Written informed consent must be obtained from the patient. Exclusion criteria (1): Individuals with organic brain lesions, intellectual disabilities, or other physical ailments (2). Those experiencing intracranial hypertension (3). Frequent or persistent migraines (4). Individuals with a personal history of epilepsy or a familial history of epilepsy (5). Patients possessing metallic implants within their bodies (6). Severe substance uses disorder (7). Pregnant women or those who are breastfeeding (8). Subjects currently displaying significant abnormalities in laboratory tests (i.e. blood routine, liver and kidney function, electrolytes, and thyroid function. Results that exceed the normal range and are recognized by the attending psychiatrist as having clinical significance, such as severe anemia, acute liver/kidney function disorders) (9). Patients undergoing modified electroconvulsive therapy (10). Individuals with a history of limb disability or leg injury. Participants will be evaluated based on the inclusion and exclusion criteria outlined in **Table 1**.

**Table 1:** World Health Organization trial registration data set related to this study.

Data category	Information
Primary Registry and Trial Identifying Number	ChiCTR2500106980
Date of Registration in Primary Registry	1. August. 2025
Secondary Identifying Numbers	N/A
Source(s) of Monetary or Material Support	National Natural Science Foundation of China (grant number 82301689); Medical Science and Technique Foundation of Henan Province (SBGJ202403043)
Primary Sponsor	Yange Wei, MD. Ph.D., The Second Affiliated Hospital of Xinxiang Medical University, Henan Mental Hospital, 207 Qianjin Road, Xinxiang 453002, Henan. China
Secondary Sponsor(s)	N/A
Contact for Public Queries	Zengyuan Shen, The Second Affiliated Hospital of Xinxiang Medical University, Henan Mental Hospital, 50250101154@stu.xxmu.edu.cn
Contact for Scientific Queries	Yange Wei, MD. Ph.D., The Second Affiliated Hospital of Xinxiang Medical University, Henan Mental Hospital, weiyange@xxmu.edu.cn
Public Title	Efficacy and mechanisms underlying MRI-guided HD-tDCS combined with aerobic exercise to ameliorate cognitive impairment associated with schizophrenia
Scientific Title	Efficacy and mechanisms underlying MRI-guided HD-tDCS combined with aerobic exercise to ameliorate cognitive impairment associated with schizophrenia
Countries of Recruitment	China
Health Condition(s) or Problem(s) Studied	Schizophrenia
Intervention(s)	High-definition transcranial direct current stimulation combined with aerobic exercise
Key Inclusion and Exclusion Criteria	Inclusion criteria: 1. The current episode of the patient aligns with the diagnostic criteria for schizophrenia according to the Diagnostic and Statistical Manual of Mental Disorders-5 (DSM-5). 2. patients who achieved the stable period through oral antipsychotic drug treatment, as judged by the following criteria: delusion, hallucinatory behaviors, exaggeration, and suspicion/victimization items in the positive and negative symptom scale (PANSS), abnormal thought content scores of ≤ 5 in the general psychopathology scale, and PANSS conceptual disorganization scores of ≤ 4, if all the aforementioned criteria were met, the patient was considered to be in a stable period of schizophrenia (45). 3. Patient must be undergoing treatment with atypical antipsychotic medications, with equivalent doses calculated using the defined daily dose method. 4. To minimize study population heterogeneity only Han ethnic participants will be enrolled. 5. Aged between 18 and 55 years. 6. Written informed consent must be obtained from the patient. Exclusion criteria: 1. Individuals with organic brain lesions, intellectual disabilities, or other physical ailments. 2. Those experiencing intracranial hypertension. 3. Frequent or persistent migraines. 4. Individuals with a personal history of epilepsy or a familial history of epilepsy. 5. Patients possessing metallic implants within their bodies. 6. Severe substance uses disorder. 7. Pregnant women or those who are breastfeeding. 8. Subjects currently displaying significant abnormalities in laboratory tests (i.e. blood routine, liver and kidney function, electrolytes, and thyroid function. Results that exceed the normal range and are recognized by the attending psychiatrist as having clinical significance, such as severe anemia, acute liver/kidney function disorders). 9. Patients undergoing modified electroconvulsive therapy. 10. Individuals with a history of limb disability or leg injury.
Study Type	Interventional Allocation: randomized Masked: double blind Primary purpose: schizophrenia intervention
Date of First Enrollment	December 2025
Sample Size	48
Recruitment Status	Pending
Primary Outcome(s)	Changes in The Chinese version of the MATRICS Consensus Cognitive Battery (MCCB)
Key Secondary Outcomes	Changes in Repeatable Battery for the Assessment of Neuropsychological Status (RBANS), Wisconsin Card Sorting Test (WCST), Facial Emotion Perception Test (FEPT), the Voice Emotion Perception Test (VEPT), Brief Psychiatric Rating Scale (BPRS), Positive and Negative Syndrome Scale (PANSS), Social Disability Screening Schedule (SDSS) and the Schizophrenia Quality of Life Scale (SQLS)
Ethics Review	Approved (Approval Number: XYEFYLL-2025-16) Approval Date: 17 February 2025 The Second Affiliated Hospital of Xinxiang Medical University, xyefyll@126.com, +86 0373-3373500
Completion date	Pending
Summary Results	Pending
IPD sharing statement	N/A

### Randomization

The randomization process will be conducted by an independent researcher who is not involved in the experiment. This researcher will utilize a random number table to randomly allocate participants into four distinct groups, labeled as “1, 2, 3, and 4.”. Specifically, the group coding is as follows: Group 1 corresponds to the HD-tDCS combined with AE group, Group 2 to the HD-tDCS group, Group 3 to the AE group, and Group 4 to the control group. The random allocation process will be implemented using Microsoft Excel 2019. Upon the recruitment of all 48 eligible participants, each individual will be assigned a unique identifier and paired with a randomly generated value using Excel’s “=RAND ()” function. The “Paste Special” function will then be employed to convert these random numbers into fixed values. Subsequently, the list of participants will be sorted in descending order based on the random values, with participants allocated to groups 1 to 4, each comprising 12 individuals, according to their ranking positions. Once the randomization scheme is established, it will be sealed within an opaque envelope, which will be opened sequentially in accordance with the order of subject enrolment. The allocation scheme contained within each envelope will dictate the group assignment for the corresponding participant. This approach ensures that researchers are unable to access the randomization scheme in advance, thereby safeguarding the integrity of the randomization process.

### Blinding

All participants and researchers will remain blinded throughout the research process. During the entire research process, the designated stimulus conditions will be kept strictly confidential and will only be disclosed to the principal researcher. Four distinct groups will be tasked with administering the treatment, with strict measures in place to prevent inter-group communication regarding the particulars of each treatment. Each group will be informed only of its own treatment protocol and will remain unaware of the grouping details or the treatment plans of the other groups. The principal investigator will assign specific measures to each group, which will then implement the experimental treatment for their respective patient cohorts. Patients will remain unaware of their group allocation and will be instructed not to discuss the grouping, the treatment received, the completion of questionnaires, or any other aspects of the experimental protocol. During the phase of result measurement, personnel involved in data collection and analysis will also be blinded to the grouping information and specific measures. Subsequently, the principal investigator will conduct two unblinding procedures: the first will occur before data locking, and the second will follow data analysis. The initial unblinding will occur after data locking, organizing the data into groups 1–4 without disclosing the actual correspondence between these groups and their respective identities. The second unblinding will take place after the data analysis, explicitly revealing the specific identities of groups 1-4. Furthermore, to ensure that participants are unaware of their group assignments, the intervention activities will be conducted in designated rooms. Although the researchers will be aware of the group assignments, they must not disclose this information to the participants and will not be involved in the evaluation or analysis of the study. Additionally, the evaluators and data analysts will remain unaware of the intervention group’s situation throughout the entire research process.

All researchers involved in data management and statistical analysis will remain blinded to the grouping information throughout the entire process. Specifically, data will be analyzed using coded group identifiers (e.g., Group A, B, and C), and the blinding code will remain undisclosed until the completion of the statistical analysis and the locking of the database. This procedure aligns with the principle of the blind method for minimizing assessment bias, as outlined in clinical trial protocols (46).

A Blinding Integrity (BI) assessment will be conducted following the final intervention session. Participants will be required to specify their perceived group allocation via a forced-choice questionnaire, asking: “Which intervention do you believe you received?” The response options will include (1): Combined HD-tDCS and AE (2), HD-tDCS alone (3), AE alone (4), Control, or (5) Unsure. To rigorously assess the blinding effect, Bang’s Blinding Index (BI) will be calculated for each group. This index allows for the distinction between genuine unblinding and random guessing. The BI is the primary tool for quantitatively evaluating the effectiveness of blinding implementation in clinical trials. By calculating the BI separately for each group, we can accurately assess the maintenance of blinding across different intervention groups (47).

Unlike drug trials, these trials—such as those involving surgery, rehabilitation, psychological, and physical therapy—cannot physically blind the “treatment itself” (48). During the informed consent process, we informed participants that the study was designed to investigate the effects of non-pharmacological interventions on cognitive function of individuals with schizophrenia. In the informed consent form, all intervention measures were described as “interventions with potential benefits,” without disclosing which group was the experimental group and which was the control group.The language used in the informed consent form was intentionally general to avoid revealing specific differences between groups, thereby minimizing the expectancy effect. Upon completion of the study, it is imperative to fully disclose the actual research design and the group assignments to all participants.

### MRI-guided high-definition transcranial direct current stimulation intervention

To construct precise and anatomically accurate head models, T1- and T2-weighted MRI data will be collected to optimize electrode placement and account for individual anatomical variations. These models will be developed and simulated using SimNIBS software to estimate the electric field distributions resulting from HD-tDCS stimulation. Eight distinct 4*1 montages centered on the mPFC will be used to simulate 280 the E-field for each brain. The finite element method will be utilized to compute the normal component of the induced electric field. The head models, derived from individual MRI data will differentiate among five tissue types: skin, skull, cerebrospinal fluid, gray matter, and white matter. The mPFC will be delineated in each participant’s brain according to the Ranta atlas. The study will utilize an MRI-guided HD-tDCS device (MxN-9-9002A, Soterix Medical, New York, USA). We used five small circular electrodes (1×1 cm each) with a small area for high-precision electrode arrangement, concentrating the stimulation current on the target brain region. Specifically, the central electrode (anode) will be placed in the mPFC (fz) according to international 10–20 system, while the four surrounding cathodes will be directly located in front of Fp1, Fp2, F7, and F8, thus forming a circular current circuit. The current intensity will be maintained at 2 mA. Each HD-tDCS session will involve the delivery of a 2mA direct current for a duration of 30 minutes, with ramp-up and ramp-down periods of 30 seconds each. HD-tDCS will be administered once daily for 30 minutes, five times a week over a total duration of 4 weeks, culminating in 20 treatment sessions.

### Aerobic exercise

AE training will be administered using a stationary bicycle. The training sessions will be characterized by moderate to low intensity and will include both a warm-up phase and a formal AE phase. Each session is customized according to each individual’s maximum heart rate (HRmax), which is calculated using the formula: HRmax = (220 - Age) × 0.7. (34). Each session will last for 45 minutes, comprising a 15-minute warm-up and a 30-minute formal AE phase. During the warm-up, participants’ heart rates are expected to remain between 55% and 60% of HRmax. In the formal AE phase, heart rates should be maintained between 60% and 70% of HRmax (34). A portable monitor will be utilized to record heart rates throughout the training, ensuring participants maintain an appropriate level of physical exertion. Blood pressure measurements will be taken both before and after the sessions. The intervention will occur once daily, 5 days per week, over a period of 4 weeks, culminating in 20 treatment sessions.

### Multimodal neuroimaging acquisitions and analyses

Structural T1-weighted MRI images will be acquired using a three-dimensional magnetization-prepared rapid gradient-echo (3D MPRAGE) sequence with the following parameters: repetition time (TR)/echo time (TE)/inversion time (TI) = 240/2.14/100 ms, flip angle = 8 degrees, field of view (FOV) = 224 x 224 mm, voxel size = 0.7 mm isotropic, bandwidth = 210 Hz/pixel, integrated parallel acquisition techniques (iPAT) = 2, and an acquisition time of 7 minutes and 40 seconds. T2-weighted images will be acquired using a 3D T2 sampling perfection with application-optimized contrasts using different flip angle evolutions (T2-SPACE) sequence, with parameters: TR/TE = 320/565 ms, variable flip angle, FOV = 224 x 224 mm, voxel size = 0.7 mm isotropic, bandwidth = 755 Hz/pixel, iPAT = 2, and an acquisition time of 8 minutes and 24 seconds. The total duration for structural imaging acquisition will be 16 minutes and 4 seconds. Resting-state functional MRI (fMRI) images will be acquired with the following specifications: TR/TE = 720/33.1 ms, flip angle = 52 degrees, FOV = 208 x 180 mm, matrix size = 104 x 90, slice thickness = 2.0 mm, 72 slices, 2.0 mm isotropic voxels, multiband factor = 8, echo spacing = 0.58 ms, and bandwidth = 229 Hz/pixel. The acquisition of resting-state functional images will require 14 minutes and 33 seconds.

A 48-channel functional near-infrared spectroscopy (fNIRS) device (NirScan model from Danyang Huichuang Medical Equipment Co. Ltd in China) will be employed in this study. The fNIRS data will be collected during the administration of The Chinese version of the Verbal Fluency Test (VFT) serves to evaluate verbal fluency, working memory, verbal recall, attention, and retrieval capabilities. The VFT task is structured into three distinct phases: a 30-second pre-task baseline period, a 60-second task period, and a 70-second post-task period. The baseline phase is characterized by the absence of VFT task performance. During the 30-second pre-task baseline phase, participants are instructed to count repeatedly from one to five until the commencement of the task period. In the subsequent 60-second VFT task period, participants are required to generate as many phrases as possible using simple words such as “white,” “north,” and “big.” Following the completion of the phrase generation task, participants are instructed to resume counting from one to five repeatedly throughout the 70-second post-task period. Fifteen light source probes and sixteen light detector probes will be positioned on the bilateral frontotemporal cortex, with a distance of 3 cm maintained between each light source and detector probe. In accordance with the 10/20 electrode placement system, the central probe will be positioned at FPz, while the lower boundary of the probe array will extend from Fp1 to Fp2. Hemodynamic changes will be assessed through measurements of oxyhemoglobin, deoxyhemoglobin, and total hemoglobin. Resting-state and task-state fNIRS data acquisitions will be performed for all participants in this study. The raw fNIRS data will be preprocessed utilizing the MATLAB Home 3 toolkit. Five statistical metrics—mean, variance, skewness, kurtosis, and peak value—will be calculated for changes in oxy-Hb signals, based on the spatial average across all 48 channels. This methodology will generate a total of 240 independent features for each subject.

The resting-state EEG data were recorded utilizing a PN-NET multichannel electroencephalogram device with 64 scalp electrodes, 1 reference electrode on the nose tip, and 2 electro-ocular electrodes positioned at the right eye corner and beneath the left eye. Data collection transpired in a tranquil examination room by a consistent researcher. Subjects were instructed to maintain stillness, wakefulness, and relaxation, minimize bodily and ocular movements, and abstain from specific tasks. Electrode impedance was maintained below 5 kΩ to ensure data fidelity during collection. A sampling rate of 1,024 Hz was employed, capturing EEG data with closed eyes over a 5-minute interval.

### Biological assessment

Participants will undergo a fasting anterior elbow vein puncture to collect a 5 ml blood sample, which will be transferred to an anticoagulant tube and centrifuged at 300 rpm for 10 minutes. The supernatant and sedimented blood cells will be separately transferred into two Eppendorf tubes. Subsequently, the centrifuge tube will be placed in a freezing centrifuge for further centrifugation. The supernatant will be aspirated, and its protein concentration will be quantified using the BCA kit. Subsequently, equal volumes of samples and molecular weight standards will be subjected to Tris-SDS-PAGE electrophoresis, followed by transfer onto a PVDF membrane. The membrane will be incubated in a 5% skimmed milk solution for blocking for a duration of 2 hours. After a washing step, primary antibodies specific to BDNF and β-actin, diluted at a ratio of 1:500, will be applied and incubated overnight at 4°C. Thereafter, secondary antibodies, also diluted at a ratio of 1:500, will be introduced and incubated at room temperature for 2 hours. The PVDF membrane containing the target protein will then be immersed in a developing solution for analysis using a chemiluminescence imaging analyzer. The exposure time will be adjusted based on the protein’s abundance, and the expression levels of BDNF will be quantitatively assessed using ImageJ software.

### Outcomes

The primary outcome measure will be the change in scores on the Chinese version of the Food and Drug Administration utilizes the MATRICS Consensus Cognitive Battery (MCCB). Assessments will be conducted at baseline (T0), at 2 weeks (T1), at 4 weeks (T2), and at 6 months post-intervention (T3). The MCCB consists of 10 cognitive tests (3), it covers a total of seven different cognitive domains, including speed of processing, attention/vigilance, working memory, verbal learning, social cognition, reasoning and problem-solving (49). MCCB provides a comprehensive evaluation of specific cognitive domains associated with schizophrenia. Assessments will be conducted at baseline (T0), at 2 weeks (T1), at 4 weeks (T2), and at 6 months post-intervention (T3).

For the assessment of secondary outcomes, this study employed the Repeatable Battery for the Assessment of Neuropsychological Status (RBANS) and the Wisconsin Card Sorting Test (WCST) to evaluate global and executive functions. Simultaneously, the Facial Emotion Perception Test (FEPT) and the Voice Emotion Perception Test (VEPT) were utilized to assess emotion perception abilities within the domain of social cognition. The severity of clinical symptoms and overall changes were quantified using the Positive and Negative Syndrome Scale (PANSS) and the Brief Psychiatric Rating Scale (BPRS). Furthermore, improvements in social functioning and quality of life were evaluated using the Social Disability Screening Schedule (SDSS) and the Schizophrenia Quality of Life Scale (SQLS). The RBANS comprises five components: immediate memory, visuospatial skills, language, attention, and delayed memory. Its notable features include ease of administration, brief administration time, and robust reliability and validity. The RBANS is effective for detecting cognitive impairment and can also be used to assess changes in cognitive function following treatment or intervention (50). The WCST is a widely utilized standardized tool in neuropsychological research and clinical cognitive assessment, primarily crafted to assess cognitive flexibility and abstract thinking within executive functions. Participants must discern classification rules based on evolving feedback (e.g., color, shape, or number) and adapt their strategies accordingly. This assessment effectively mirrors the operation of the prefrontal cortex and is frequently employed to pinpoint executive function impairments linked to psychiatric disorders, brain injuries, and ageing (51). The FEPT is predominantly employed to gauge an individual’s capacity to perceive and comprehend the emotions conveyed in the facial expressions of others. This evaluation presents a sequence of standardized facial expression images (e.g., happiness, sadness, anger, fear, etc.), requiring participants to recognize the type or intensity of emotion (52). The VEPT is employed to assess an individual’s ability to perceive emotional nuances in others’ voices. Participants listen to voice samples (neutral or expressing emotions like joy, sadness, anger, etc.) and must identify the specific emotion conveyed (53). The BPRS is utilized to gauge the intensity of psychiatric symptoms, with higher scores indicating more pronounced symptom severity (54). The PANSS is a widely used standardized tool in psychiatric clinical and research settings, primarily crafted to evaluate symptom severity in individuals with schizophrenia (55). It consists of the Positive Symptom Scale and the Negative Symptom Scale, each comprising 7 items, alongside a general psychopathology scale with 16 items, totaling 30 items. The SDSS is employed to quantitatively evaluate social functioning impairment in patients, encompassing crucial aspects such as work and social interactions. It is user-friendly and suitable for rehabilitation assessments (56). The SQLS assesses patients’ quality of life using various indicators that mirror their subjective experiences, offering a benchmark for evaluating effectiveness (57).

To explore the underlying mechanisms, the study adopted a multimodal assessment framework. MRI and fNIRS were utilized to investigate changes in brain activity, structure, and functional connectivity. EEG and P300 were employed to capture the neurophysiological dynamics of electrical brain activity. EM served as an objective behavioral-physiological marker of cognitive processing. Additionally, plasma BDNF levels were measured to evaluate the molecular correlates of neurotrophic support and plasticity. This integrated approach aims to elucidate the therapeutic effects of MRI-guided HD-tDCS combined with AE for CIAS and to uncover the neurobiological mechanisms underlying these effects.The procedure for the site visit is outlined in **Table 2**.

**Table 2:** Time schedule of screening, interventions and assessments.

Item	Recruitment	Assessment time point			
		T0	T1	T2	T3
Prescreening for eligibility, consenting, and clinical interview							
Recruitment					
Eligibility screening	✓				
Informed consent	✓				
Allocation	✓				
Primary outcome assessment					
MCCB		✓	✓	✓	✓
Second outcome assessment					
RBANS		✓	✓	✓	✓
BPRS		✓	✓	✓	✓
SQLS		✓	✓	✓	✓
SDSS		✓	✓	✓	✓
PEPT		✓	✓	✓	✓
VEPT		✓	✓	✓	✓
WCST		✓	✓	✓	✓
Biological assessment					
fNIRS		✓	✓	✓	✓
EEG		✓	✓	✓	✓
EM		✓	✓	✓	✓
P300		✓	✓	✓	✓
MRI		✓	✓	✓	✓
Plasma BDNF		✓	✓	✓	✓
Safety					
ARS			✓	✓	✓

A checkmark (✓) indicates the time point at which each assessment is carried out. ARS, Adverse Reaction Scale; BPRS, Brief Psychiatric Rating Scale; EEG, Electroencephalography; EM, Eye Movements; fNIRS, Functional Near-Infrared Spectroscopy; MCCB, MATRICS Consensus Cognitive Battery; MRI, Magnetic Resonance Imaging; PANSS, Positive and Negative Syndrome Scale; PEPT, Facial Emotion Perception Test; P300, P300 Event-Related Potential; RBANS, Repeatable Battery for the Assessment of Neuropsychological Status; SDSS, Social Dysfunction Screening Scale; SQLS, Schizophrenia Quality of Life Scale; VEPT, Voice Emotion Perception Test; WCST, Wisconsin Card Sorting Test.

T0: baseline, T1: after 2 weeks, T2: after 4 weeks, T3: after 6 months.

### Sample size calculation

We performed the necessary calculations using G*Power software (version 3.1.9.7). Under the following command, test family: F tests; Statistical test: ANOVA: Repeated measures, within-between interaction (58).The research parameters were defined as follows: a Type I error rate of 0.05, a statistical power of 80%, and an effect size of 0.25. Utilizing a repeated measures ANOVA model and considering a potential participant dropout rate of 20%, we determined that the required sample size is 48 patients, with 12 patients assigned to each group. In preliminary studies where prior sample size data are unavailable, a group size of 12 subjects is deemed appropriate. We will justify the rationale for selecting a sample size of 12 per group from three perspectives: feasibility, improved precision in estimating means and variances, and adherence to regulatory requirements (59). This sample size is expected to provide adequate statistical power to address the study objectives.

### Date management

The evaluation of cognitive function and the severity of psychiatric symptoms will be conducted by two psychiatrists, who will remain blinded to the group assignments. All demographic information and scale-related data will be recorded in electronic Case Report Forms (eCRFs) and stored on a designated website. Access to the securely stored eCRFs will be restricted to the project leader and the principal investigator. To maintain confidentiality, anonymization will be implemented during data entry by substituting patients’ actual names with unique identification numbers. The independent data monitoring committee will be established to monitor safety, the occurrence of adverse events, and advise on trial design decisions.

### Statistical methods

The Shapiro-Wilk test will be employed to determine whether the quantitative data conform to a normal distribution. When the quantitative data are normally distributed, they will be described using the mean and standard deviation. If the data deviate from a normal distribution, the median and interquartile range will be utilized for presentation. Categorical data will be presented as counts and percentages. To evaluate differences in baseline characteristics among groups, appropriate statistical tests such as t-tests, nonparametric tests, chi-square tests, Mann-Whitney U tests, or ANOVA will be selected based on the type and distribution of the data. Additionally, Fisher’s exact test or chi-square test will be used to compare adverse reactions between groups.

**Figure 2:**
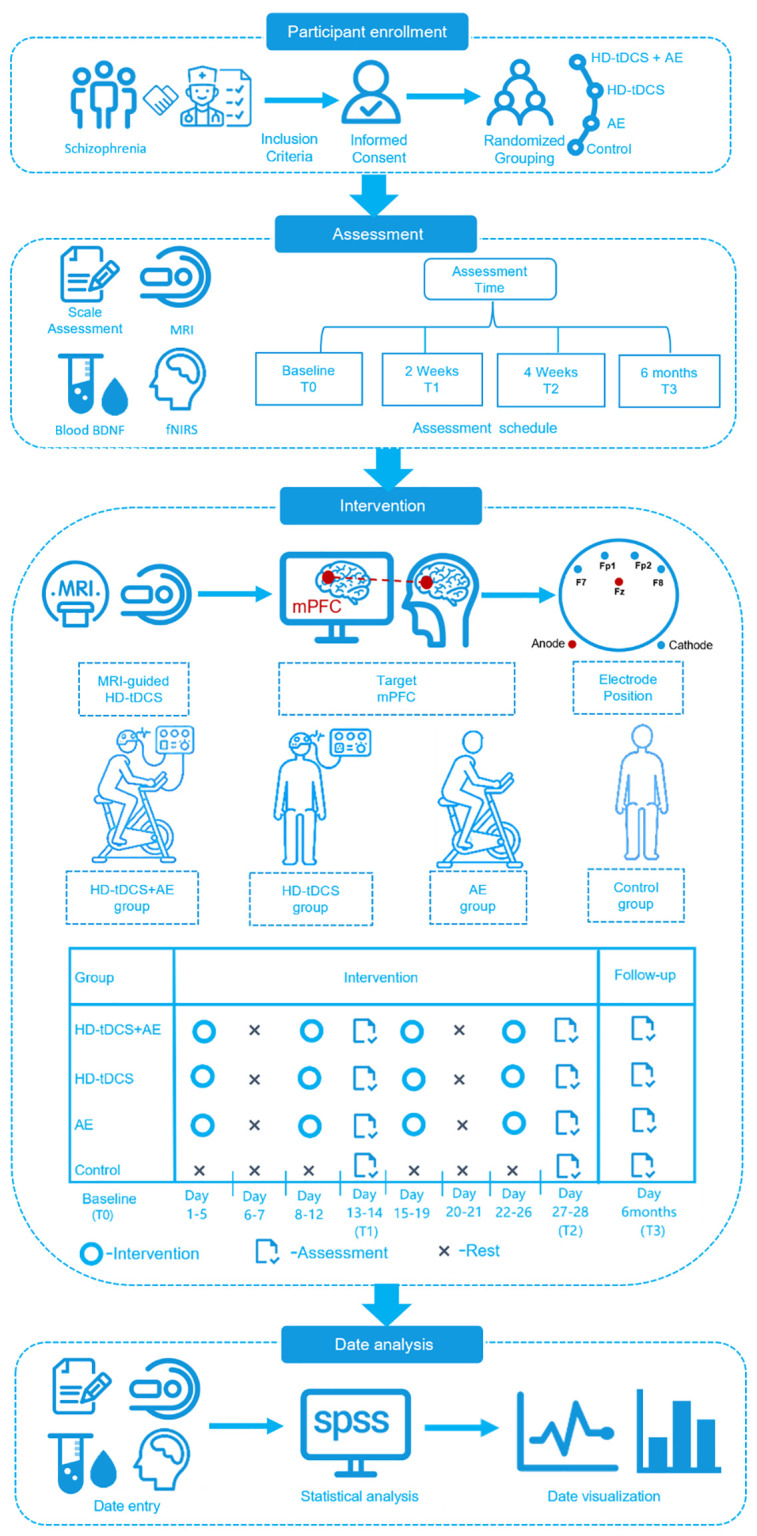
Study schedule for the participants in this randomized, double-blind, controlled trial. AE, Aerobic Exercise; fNIRS, Functional Near-Infrared Spectroscopy; HD-tDCS, High-definition transcranial direct current stimulation; MRI, magnetic resonance imaging.

This study will adhere to the CONSORT guidelines and perform data analysis based on the intention-to-treat principle, ensuring that all participants are evaluated according to their initial randomization assignments. To assess differences in primary and secondary outcomes both between and within groups over time, generalized linear mixed models and repeated-measures ANOVA will be employed as appropriate. A stepwise model selection approach will be implemented to derive a succinct multivariate regression model. Age, gender, and the Defined Daily Dose (DDD) of antipsychotic medications will be included as covariates in all models. The treatment effect will be evaluated using the likelihood ratio test to ascertain whether the coefficients for treatment and the interaction between time and treatment are both zero. To correct for multiple comparisons across time points, the Bonferroni correction method will be applied to adjust the p-values. All statistical analyses will be conducted using SPSS version 20.0, with a significance threshold set at *P* < 0.05 to denote statistical significance. Research framework is illustrated in **Figure 2**.

### Data monitoring

Despite being deemed low-risk for participants, the Institutional Review Board of the Second Affiliated Hospital of Xinxiang Medical University opted to form a data monitoring committee for the ensuing experiments to safeguard data quality and participant well-being.

### Harms

To evaluate the safety and potential adverse effects of HD-tDCS, this study will thoroughly investigate both severe and mild adverse events. All participants will be required to complete the HD-tDCS Adverse Reaction Scale (ARS), which encompasses common side effects such as tingling, mild redness, itching, and discomfort at the stimulation site. Participants will rate their adverse reactions on a scale from 0 to 5, with all occurrences documented in the CRFs. The HD-tDCS equipment will be operated by trained therapists proficient in its use, aiming to minimize the risk of participants encountering any significant health hazards or adverse effects. In the event of a severe adverse reaction, the participant will be withdrawn from the study, and the incident will be promptly reported to the ethics committee.

### Auditing

To ensure data quality, a dual verification system will be implemented throughout the study, covering both the treatment phase and the 6-month follow-up period. The lead researcher will conduct weekly evaluations of CRFs and estimation forms. Additionally, a bi-weekly comparison between hard copy and electronic records will be performed to verify consistency. Any discrepancies will be documented and discussed in team meetings to facilitate prompt corrective actions.

### Protocol modifications

The procedure for implementing substantial protocol modifications requires submitting all changes to the Ethics Review Committee of the Second Affiliated Hospital of Xinxiang Medical University for approval. Upon receiving approval, the research team will formally notify all relevant stakeholders in writing. Subsequently, the approved amendments will be promptly recorded on the Chinese Clinical Trial Registry platform.

### Dissemination policy

Prior to enrolling any participants, the research team will register and disclose the trial details on the Chinese Clinical Trial Registry. Furthermore, there is a commitment to publicly release the complete study outcomes. The dissemination of results will be achieved through presentations at international conferences and submissions for peer-reviewed publication in scientific journals.

### Confidentiality

Regarding confidentiality, all study personnel have received training and certification in human subjects’ research protections through the Second Affiliated Hospital of Xinxiang Medical University Training Program. The Principal Investigator provides ongoing training and oversight to the evaluation coordinator to maintain the confidentiality and privacy of all participants and their data.

### Data sharing

Raw data will be generated at the Second Affiliated Hospital of Xinxiang Medical University. Derived data supporting the findings of this study will be will be accessible upon request from the corresponding author.

## Discussion

To the best of our knowledge, this may represent the first randomized, double-blind, controlled clinical trial investigating the potential of combining MRI-guided HD-tDCS with AE to enhance cognitive function for CIAS at the individual level. Most non-pharmacological interventions focus exclusively on either HD-tDCS or AE. HD-tDCS has been demonstrated to enhance executive function and working memory by modulating neuronal excitability in the mPFC (60). Meanwhile, AE influences brain plasticity, metabolism, structure, and connectivity by promoting neurogenesis and structural changes, thereby improving cognitive function (28, 61). Here in, we aim to integrate these two non-pharmacological approaches to examine their synergistic effect on cognitive enhancement for CIAS. This synergistic intervention strategy may break through the limitations of single intervention and provide aa multi-dimensional view of cognitive improvement. This could eventually provide individualized precision treatment strategies to increase treatment effectiveness.

This prospective, randomized, double-blind, controlled study will utilize MRI-guided HD-tDCS targeting mPFC to improve cognitive function for CIAS. Considering the significant impact of inter-individual neuroanatomical differences on HD-tDCS outcomes, MRI was employed to precisely identify and localize the target region for each participant. This customized approach ensured accurate electrode positioning, thereby enhancing stimulation precision and efficacy. Previous HD-tDCS research has demonstrated significant improvements in cognitive symptoms, particularly in executive functions (13, 62, 63). Given its role in advanced cognitive regulation, decision-making, working memory, and emotion control (64), the mPFC is a crucial brain region involved in the pathophysiology of schizophrenia. Its functional abnormalities are associated with CIAS, including impairments in working memory and attention control (65). Autopsy studies have demonstrated a significant reduction in the density of dendritic spines within the mPFC neurons of patients with schizophrenia (21). Studies utilizing animal models have revealed that abnormal dopaminergic regulation of the mPFC contributes to cognitive impairment (19). Investigations employing conventional HD-tDCS have indicated that stimulation of the mPFC can enhance language fluency and working memory in patients (66). Consequently, the mPFC may serve as a potential target for CIAS.

AE as an adjunctive intervention for chronic schizophrenia, may improve cognitive functions across five domains: information processing speed, working memory, problem-solving skills, verbal learning and memory, and visual learning and memory, consistent with previous research findings (33). Learning and memory functions are associated with the mPFC and hippocampus (67). Individuals with schizophrenia exhibit irregularities in neural growth and neurotransmitter release in mPFC (40), as well as alterations in hippocampal volume and morphology (68, 69). AE has been shown to improve mitochondrial structure and function in the mPFC, increase the release of neurotrophic factors such as BDNF (38), enhances aerobic metabolism (70), and mitigates hypoxia and inflammation (43, 71). AE interventions contribute to increased hippocampal volume in individuals with schizophrenia (72, 73), as well as elevated levels of oxygenated hemoglobin in the mPFC, which are closely linked to improved executive function (28). Animal studies using schizophrenia models further confirmed that exercise enhances hippocampal volume and cognitive abilities (73). Collectively, these findings imply that AE may improve cognition by modulating brain function and neurotransmitter release in the mPFC, which are critical for cognitive processes.

This study explores the integration of MRI-guided HD-tDCS with AE to enhance cognitive function in individuals with CIAS. This combined approach holds potential for mitigating adverse drug reactions, such as extrapyramidal symptoms and endocrine disorders, thereby improving patients’ overall quality of life. In other words, this approach provides a scalable non-pharmacological intervention, particularly advantageous for patients who experience limited drug efficacy or intolerable side effects. AE could simultaneously enhance cerebral blood flow and elevate neurotrophic levels, and achieve a synergistic effect when combined with electrical stimulation. This application could amplify therapeutic outcomes through a synergistic process. HD-tDCS can be safely administered in domestic settings, eliminating the need for frequent hospital visits and thereby reducing time and transportation costs. The flexibility of AE allows patients to select exercise methods tailored to their individual conditions, potentially improving adherence to interventions among individuals with CIAS and facilitating long-term treatment possibilities. In comparison to more complex neuroregulation techniques such as transcranial magnetic stimulation, this integrated strategy may overcome the limitations of single interventions. In the long term, it demonstrates high cost-effectiveness and is particularly suitable for implementation in community settings and low-income regions.

In this study, we will employ multi-dimensional measures to investigate the biological mechanisms of HD-tDCS combined with AE in individuals with CIAS. With the progress in individualized precision medicine, researchers are seeking biomarkers to guide therapy or access patient outcomes in schizophrenia. MRI data will be utilized to determine the optimal electrode placement, accounting for individual anatomical variations, and to evaluate alterations in brain structure and function. The HD-tDCS will target the mPFC to modulate neuronal excitability, with MRI employed to assess potential morphological enhancements in this region following the intervention, thereby providing structural-level support for cognitive enhancement. fNIRS will be used as a direct measure of neural activity to evaluate both local and network effects (74–76). Due to its high temporal resolution, robust resistance to motion artifacts, and cost-effectiveness (77), fNIRS is well-suited for the dynamic assessment of changes in brain function during interventions. Previous research has demonstrated a significant reduction in oxygenated hemoglobin signals within the mPFC during VFT in individuals with schizophrenia, which was negatively associated with cognitive performance (78). This study will utilize fNIRS to quantitatively evaluate the activation patterns of the mPFC, thereby providing objective insights into the neural mechanisms underlying a combined intervention. Previous research has demonstrated that AE can increase BDNF levels (38). Furthermore, P300, and EEG will be employed as objective measures to deliver a more comprehensive and systematic analysis of the neural mechanisms involved in the combined intervention. We hypothesize that the combined strategy may induce significant changes in mPFC activity and BDNF levels. We also hypothesize that these changes will correlate with improvements in cognitive function. If so, the study will provide objective evidence supporting the efficacy of HD-tDCS combined with AE, underscoring its potential as an individualized therapeutic strategy for addressing CIAS at the biological level.

This study presents some limitations. Firstly, the lack of a “sham” exercise or “sham” HD-tDCS compromises the complete blinding of participants, which may enhance the “expectancy effect” within the combined group. Although this study primarily investigates the synergistic feasibility of the two active interventions, future research should incorporate Sham-tDCS and an Active Control condition (such as stretching or low-intensity tasks) to balance “attention time” and “treatment expectation” across all participant groups. Secondly, cognitive tasks during treatment were not included in this study. Future research should incorporate cognitive tasks (such as N-back) concurrently with the intervention measures to enhance the magnitude and specificity of cognitive improvement. Thirdly, the sample size is relatively small, potentially compromising statistical power. Fourthly, the study lacks an analysis of various parameters of AE, Consequently, future research should focus on enlarging the sample size, and investigating optimal AE parameters. Fifthly, this study is the single center design, which may have a center effect and a selection bias. Further larger, prospective, multicenter studies are worthwhile.

This randomized, double-blind, controlled clinical design incorporated a multi-dimensional assessment will provide biological insights for the synergistic effect of HD-tDCS and AE on cognitive improvement. The integration of MRI-guided HD-tDCS targeting the mPFC with AE constitutes a promising avenue for cognitive rehabilitation in schizophrenia. This approach holds significant translational and clinical potential in precision psychiatry. Implementing these individualized therapeutic strategies may yield substantial clinical and economic benefits in the management of CIAS.
