# Supplementary material for: Efficacy and mechanisms underlying MRI-guided HD-tDCS combined with aerobic exercise to ameliorate cognitive impairment associated with schizophrenia
Source: Front Psychiatry. 2026 Feb 24;17:1742634. doi: 10.3389/fpsyt.2026.1742634 (PMC12971684; doi:10.3389/fpsyt.2026.1742634)
Supplement: Supplementary file 1 [file Supplementaryfile1.docx]

**Supplementary Material**

**Informed Consent Form**

**1. Introduce**

We would like to submit the manuscript entitled " Efficacy and mechanisms underlying MRI-guided HD-tDCS combined with aerobic exercise improves cognitive function in schizophrenia: a randomized, double-blind, sham-controlled clinical trial" for publication in ***Frontiers in Psychiatry.***

Cognitive impairment is the core symptom of schizophrenia, the cognitive function of individuals with schizophrenia does not necessarily improve with the remission of psychotic symptoms. In addition, antipsychotic medications have limited impact on the core cognitive deficits. Existing antipsychotic drugs primarily act on the dopamine system and are effective for improving positive symptoms, but their efficacy in treating negative symptoms and cognitive impairments is very limited. They also come with a range of side effects, such as extrapyramidal symptoms, metabolic syndromes, and endocrine disorders. Additionally, there are issues of individual differences in treatment response and compliance with the therapy. Therefore, we present an innovative clinical trial to investigate the potential enhancement of cognitive improvement in schizophrenia through the combination of HD-tDCS and AE. Given the potential of these two non-pharmaceutical treatments, we would like to introduce this research to you.

**2. The name of the study and approval for its implementation**

The title of this study is " Efficacy and mechanisms underlying MRI-guided HD-tDCS combined with aerobic exercise improves cognitive function in schizophrenia: a randomized, double-blind, sham-controlled clinical trial”. The study was approved by the Research Ethics Committee of the Second Affiliated Hospital of Xinxiang Medical University (Approval Code: XYEFYLL-2025-16, Approval Date: 17 February 2025). Recruitment began in September 2025 and registered with https://www.chictr.org.cn/ under protocol registration number ChiCTR2500106980 (date of registration: 1. August. 2025).

**3. Objective**

This study aims to explore three objectives. The first objective is to evaluate the improvement effect of the combined intervention of HD-tDCS and AE on the cognitive function of patients with schizophrenia. The second objective is to assess the long-term effect of the combined intervention of HD-tDCS and AE after 6 months. The third objective is to use MRI, Functional near-infrared spectroscopy (fNIRS), and serum brain-derived neurotrophic factor (BDNF) levels to explore the biological mechanism of MRI-guided HD-tDCS and AE in schizophrenia.

**4. MRI-guided HD-tDCS intervention**

The HD-tDCS is a non-invasive, effective, and cost-effective noninvasive neuromodulatory strategy for schizophrenia. MRI-guided HD-tDCS intervention can select the optimal electrode placement solution for each individual, reducing the impact of individual brain structural differences and maximizing the HD-tDCS effect. MRI-guided HD-tDCS can improve short-term memory and attention in patients with schizophrenia, enhance social cognition, and its safety has been confirmed with minimal side effects.

**5. Aerobic exercise**

Aerobic exercise (AE)has been proven to be a non-pharmacological intervention option for schizophrenia. AE can serve as a valuable adjunctive intervention for schizophrenia, especially in alleviating cognitive impairments and promoting overall health through its synergistic effects. It can serve as a valuable adjunctive treatment for schizophrenia, particularly in alleviating cognitive deficits and promoting overall well-being through its synergistic effects.

1. **Inclusion and exclusion criteria**
   1. **Inclusion criteria**
2. The current episode of the patient conforms to the clinical diagnostic criteria for schizophrenia specified in the DSM-5.
3. After taking oral antipsychotic medications, the patient's schizophrenia has reached a stable state, as evaluated by the following standards: on the Positive and Negative Syndrome Scale (PANSS), the scores for delusions, hallucinatory behaviors, exaggeration, and suspicion/victimization meet the requirements; the score of abnormal thought content on the general psychopathology scale is ≤5; and the score of conceptual disorganization on the PANSS is ≤4.
4. The patient is currently being treated with atypical antipsychotic medications, and the equivalent doses of antipsychotic drugs are calculated by the defined daily dose method.
5. The patient's age ranges from 18 to 55 years old.

The patient has provided written informed consent.

- 1. **Exclusion criteria**

1. Individuals with organic brain lesions, intellectual disabilities, or other physical ailments.
2. Those suffering from intracranial hypertension.
3. People experiencing frequent or persistent migraines.
4. Individuals with a personal history of epilepsy or a family history of epilepsy.
5. Patients who have metallic implants in their bodies.
6. Those with severe dependence on drugs and alcohol.
7. Pregnant women or women who are breastfeeding.
8. Subjects currently presenting significant abnormalities in laboratory tests.
9. Patients currently receiving Modified Electroconvulsive Therapy.
10. Individuals with a history of limb disability or leg injury.

**7. Intervention plan**

**7.1 MRI-guided HD-tDCS intervention plan**

The HD-tDCS intervention is administered once a day, for 30 minutes each time, 5 days a week, for a total of 4 weeks, with a total of 20 intervention sessions. For the purpose of the study, you will be randomly assigned to either the active HD-tDCS alone group or HD-tDCS combined with AE the group.

**7.2 Aerobic exercise**

The AE intervention is conducted once a day, each session lasting approximately 45 minutes, for 5 times a week, it lasts for 4 weeks, with a total of 20 intervention sessions. After the joint group completed a 15-minute warm-up exercise, they simultaneously underwent HD-tDCS and AE for 30 minutes each, with one session per day for a total of 45 minutes, five times a week, for a total of 4 weeks, amounting to 20 sessions in all. For the purpose of the study, you will be randomly assigned to either the AE alone group or HD-tDCS combined with AE the group.

**7.3 Control plan**

If you are assigned to the control group, you will continue with your daily medication without any intervention of HD-tDCS or AE. The following outlines how the study will be administered:

1. You will be screened to determine if you meet the inclusion criteria and do not meet the exclusion criteria.
2. Once confirmed to participate, you will be randomly assigned to one of the following groups: the Hd-tDCS+ AE group, HD-tDCS group, AE group, or the control group. The assessors conducting the evaluations and yourself will not know which group you belong to until the study is completed. Before the first stimulation, we will collect your demographic data and conduct a baseline assessment.
3. The intervention will be carried out according to the plan of the assigned group.

During the study, you will need to report any adverse reactions.

1) You will be reassessed after the 10th and 20th intervention sessions.

Please note that the study will be discontinued if any of the following conditions occur:

2) Serious adverse events and side effects emerge, making it difficult to continue the study;

3) The patient or their family is unwilling to continue participating in the trial and withdraws the informed consent.

**7.4 Outcome assessment**

Assessments will be conducted at baseline (T0), after 2 weeks (T1), after 4 weeks (T2), and after 6 months post-study (T3).

The primary outcome measure is the change in

1. MATRICS Consensus Cognitive Battery (MCCB)
2. Repeatable Battery for the Assessment of Neuropsychological Status (RBANS)

The second outcome measures include:

1. Brief Psychiatric Rating Scale (BPRS)
2. Positive and Negative Syndrome Scale (PANSS)
3. Magnetic resonance imaging (MRI
4. Functional near-infrared spectroscopy (fNIRS)
5. Clinical Global Impressions (CGI),
6. Schizophrenia Quality of Life Scale (SQLS),
7. Social Disability Screening Schedule (SDSS),
8. Facial Emotion Perception Test (FEPT),
9. Voice Emotion Perception Test (VEPT)
10. Electroencephalography (ECG)
11. General Information Questionnaire (GIQ),
12. Wisconsin Card Sorting Test (WCST)
13. Eye Movements (EM),
14. P300 Event-Related Potential (P300)
15. Brain-Derived Neurotrophic Factor (BDNF)

**8 What do you need to note?**

1) Provide accurate information about your medical history and current health status.

2) Inform the research physician of any discomfort you experience during this study.

3) Inform the research physician if you have participated in any other studies recently or are currently involved in other research.

4) Please adhere to the corresponding requirements of the study (such as not discussing with other participants what interventions you have undergone, etc.).

**9 The risk**

1) You may encounter the following risks: adverse reactions from the treatment, which may include pain, itching, mild transient skin redness, discomfort in the stimulated area, moderate fatigue, difficulty concentrating, and headaches.

2) You may experience psychological discomfort during the assessment process and distress

from being asked sensitive questions.

These are common risks in randomized clinical trials, but your information will be kept strictly confidential. If you are unwilling to bear the risks associated with being assigned to the control group (those who do not receive the intervention), you can refuse to participate. In the event of serious adverse effects, appropriate medical and nursing care will be provided until the symptoms subside, and you will not be charged any fees.

**10 Benefits**

1) You will not be required to pay any costs associated with this trial.

2) The study will provide you with feedback on your positive and negative symptoms, cognitive status, and other assessments and examinations, helping you gain a better understanding of yourself.

3) Participating in this study may accelerate your recovery from psychiatric symptoms and cognitive functions, among other conditions.

**11 Withdrawal of consent after participation**

Your participation in this study is entirely voluntary. You may refuse to participate, and declining to participate or withdrawing from the study at any time will not affect your current treatment, legal status, social services, or rights. There are two situations in which you might withdraw from the study early: one is if your doctor decides to terminate your participation for your benefit; the other is if you do not fully comply with the study protocol, in which case the researchers may also decide to discontinue your participation.

**12 If you have any questions about this research**

If you have any questions about this study, please contact our researchers:

Zengyuan Shen Email: 50250101154@stu.xxmu.edu.cn

Shanyuan He Email: 50240101132@stu.xxmu.edu.cn

However, we may not be able to respond to you or answer your questions when they involve the privacy of other participants or intellectual property rights.

**13 The system of this study**

Name of the medical institution: Department of Psychiatry, The Second Affiliated Hospital of Xinxiang Medical University.

Principal Investigator names: Yan Ge Wei, MD

**Signing of Informed Consent Form**

 I have read this informed consent form.

 I have had the opportunity to ask questions and all my questions have been answered.

 I understand that participation in this study is voluntary.

 I can choose not to participate in this study, or withdraw at any time by notifying the researcher without discrimination or retaliation, and my medical treatment and rights will not be affected.

 If I need other treatments, or if I do not follow the research plan, or if there is any research-related injury or for any other reason, the research physician can terminate my continued participation in this study.

 I decide to consent to participate in this study.

Subject's Signature: Date: ______ year ______ month ______ day

Researcher's statement: I have accurately informed the subject of this document, he/she has read this informed consent form accurately, and I certify that the subject has had the opportunity to ask questions. I certify that he/she is voluntarily consenting.

Researcher's Signature: Date: ______ year ______ month ______ day
